# Comparison of Tenofovir, Zidovudine, or Stavudine as Part of First-Line Antiretroviral Therapy in a Resource-Limited-Setting: A Cohort Study

**DOI:** 10.1371/journal.pone.0064459

**Published:** 2013-05-14

**Authors:** Kavindhran Velen, James J. Lewis, Salome Charalambous, Alison D. Grant, Gavin J. Churchyard, Christopher J. Hoffmann

**Affiliations:** 1 The Aurum Institute, Johannesburg, South Africa; 2 London School of Hygiene and Tropical Medicine, London, United Kingdom; 3 Division of Infectious Diseases, Johns Hopkins University School of Medicine, Baltimore, Maryland, United States of America; Commissariat a l'Energie Atomique(cea), France

## Abstract

**Background:**

Tenofovir (TDF) is part of the WHO recommended first-line antiretroviral therapy (ART); however, there are limited data comparing TDF to other nucleoside reverse transcriptase inhibitors in resource-limited-settings. Using a routine workplace and community-based ART cohort in South Africa, we assessed single drug substitution, HIV RNA suppression, CD4 count increase, loss-from-care, and mortality between TDF, stavudine (d4T) 30 mg dose, and zidovudine (AZT).

**Methods:**

In a prospective cohort study we included ART naïve patients aged ≥17 years-old who initiated ART containing TDF, d4T, or AZT between 2007 and 2009. For analysis of single drug substitutions we used a competing-risks time-to-event analysis; for loss-from-care, mixed-effect Poisson modeling; for HIV RNA suppression, competing-risks logistic regression; for CD4 count slope, mixed-effects linear regression; and for mortality, proportional hazards modeling.

**Results:**

Of 6,196 patients, the initial drug was TDF for 665 (11%), d4T for 4,179 (68%), and AZT for 1,352 (22%). During the first 6 months of ART, the adjusted hazard ratio for a single drug substitution was 2.3 for d4T (95% confidence interval [CI]: 0.27, 19) and 5.2 for AZT (95% CI: 1.1, 23), compared to TDF; whereas, after 6 months, it was 10 (95% CI: 5.8, 18) and 4.4 (95% CI: 2.5, 7.8) for d4T and AZT, respectively. Virologic suppression was similar by agent; however, CD4 count rise was lowest for AZT. The adjusted hazard ratio for loss-from-care, when compared to TDF, was 1.5 (95% CI: 1.1, 1.9) for d4T and 1.2 (95% CI: 1.1, 1.4) for AZT. The adjusted hazard ratio for mortality, when compared to TDF, was 2.7 (95% CI: 2.0, 3.5) and 1.4 (95% CI: 1.3, 1.5) and for d4T and AZT, respectively.

**Discussion:**

In routine care, TDF appeared to perform better than either d4T or AZT, most notably with less drug substitution and mortality than for either other agent.

## Background

Decisions on first-line combination antiretroviral therapy (ART) regimens for use in resource-limited-settings are made based on trade-offs regarding cost, need for laboratory monitoring, severity and frequency of side effects, and effectiveness. Stavudine (d4T) was initially recommended by the World Health Organization (WHO) largely due to less need for laboratory monitoring and a lower price than zidovudine (AZT) [1], [2]. As a result of subsequent accumulated experience with d4T, especially when using 40 mg dosing for individuals weighing more than 60 kg, the frequency of severe d4T related side effects became clear. As a result, the WHO recommended a reduction in d4T dose for patients weighing more than 60 kg followed thereafter with guidelines recommending tenofovir disoproxil fumarate (TDF) as part of the preferred regimen, with AZT as an alternative [3], [4].

Despite these changes in WHO recommendations, d4T and AZT remain widely used due to the higher cost of a month supply of TDF compared to either d4T or AZT. An additional limiting factor for TDF uptake is concern related to monitoring patients receiving TDF for renal toxicity, a potential severe side effect of TDF. However, in addition to the direct cost of the medication, other factors need to be considered when selecting an agent for long-term use, such as the costs of monitoring, need for managing common toxicities, frequency of the need to change regimens, and the impact, from a public health standpoint, of loss of patients from care related to medication side effects. Thus outcome assessments comparing agents during routine patient care can be valuable for informing policy regarding ART agent selection.

Two studies set in resource-limited-settings and using routine program data have compared TDF, d4T (30 mg), and AZT-based regimens. One was a study from Zambia that compared single drug substitutions and a composite mortality and loss-from-care endpoint [5]. The other was a study from Lesotho that assessed single drug substitutions and all-cause mortality [6]. These two studies provide important evidence of improved outcomes, especially fewer switches for toxicity, with the use of TDF when compared to either d4T or AZT. However, there remains room for further comparisons of TDF, d4T 30 mg, and AZT, especially using longer term data and including virologic response, and robust loss-from-care outcomes. Using a large community and workplace HIV care program, we have compared the agents TDF, d4T, and AZT on the following outcomes: single drug substitutions, HIV RNA suppression, CD4 count increase, loss-from-care, and mortality.

## Methods

### Ethics Statement

All data were analyzed anonymously and all aspects of this study were conducted according to the principles expressed in the Declaration of Helsinki. Human subject research approval for this study was received from the University of KwaZulu-Natal Biomedical Research Ethics Committee and the Johns Hopkins Institutional Review Board.

### Population

The study population consisted of HIV-infected patients enrolled in community or workplace HIV care programs applying standardized treatment and monitoring approaches and overseen by a single HIV management organization [7], [8]. The workplace program used a regimen of AZT, lamivudine (3TC), and efavirenz (EFV) or nevirapine (NVP) until mid-2007, when there was a shift to TDF, 3TC or emtricitabine (FTC), and EFV or NVP. The community program used a regimen of d4T, 3TC, and NVP or EFV until 2009 at which time the regimen shifted to TDF, 3TC, and NVP or EFV. Patients in the community program prior to 2009 could only be placed on TDF after review of a motivation from the providing clinician. From June 2007 onwards, a uniform dose of d4T of 30 mg twice daily was used, without weight-based dosing, through-out the programs. Patients in the workplace program were started on ART if they had a CD4 count less than 250 cells/mm^3^ or a CD4 count less than 350 cells/mm^3^ with WHO clinical stage III or any CD4 and WHO clinical stage IV disease; whereas the community program required a CD4 count less than 250 cells/mm^3^ or any CD4 with WHO clinical stage IV disease. Other patient monitoring and management was similar between the two programs with similar use of guidelines and program monitoring and evaluation activities.

For this study, we included patients who were ART naïve at study entry, ≥17 years old, initiated ART between June 1, 2007 and June 30, 2009, and were initiated on a regimen of TDF, d4T (30 mg twice daily), or AZT with 3TC or FTC and either EFV or NVP. Patients were excluded if they received 40 mg of d4T or if the dose was not recorded. Entry into observation was defined as the date of ART initiation. Exit was defined as the earliest date amongst patient death, time of change of any ART agent, or 24 months elapsed on ART. The study period closed on June 30, 2011, six months prior to the cohort closure, to allow for a potential 24 months of follow-up with an additional six months for assignment of appropriate loss to follow-up status for all participants.

Patient deaths were identified through clinical records and linkage with the South African National vital status registry for patients with recorded national identification numbers. We used inverse probability weighting to adjust for under-ascertainment of death among patients without recorded national identification numbers who were lost from care, up weighting individuals lost from care who had identification numbers and down weighting those lost from care who did not have identification numbers [9]. To most accurately attribute death to the correct regimen, we extended the observation time by three months from a change in an ART agent, as deaths within days of a change in ART agent would be unlikely to be associated with the new agent but could plausibly be associated with the prior regimen.

Loss-from-care was defined as any patient whose last clinic or laboratory record was before closure of the database for the specific site, in the absence of a recorded treatment stop reason or death.

### Analysis

We compared patient characteristics at ART initiation by initial NRTI (TDF, d4T, or AZT) using chi-squared or Kruskal-Wallis testing. We performed time to event analysis for each of single-agent substitution, loss-from-care and mortality. We used single agent substitution as a proxy for severe side effects, assessing risk of substitution using a competing-risks time-to-event analysis, with death and loss-from-care both considered competing risks, with robust estimates at site level. In evaluating HIV RNA suppression, we assessed the proportion with HIV RNA <400 c/mL at 24 months. We allowed for a window of 3 months earlier or later (21–27 months) and only included patients with at least one HIV RNA result during that window. We used the minimum HIV RNA value if multiple results were available during the window. We used a random-effects logistic regression for HIV RNA suppression accounting for the random effect of workplace versus community HIV program. We also assessed CD4 change over time on ART up to 24 months on ART. We only included participants with more than 12 months of follow-up to maintain consistency with the HIV RNA suppression analysis. We used mixed effects linear regression to estimate the slope in CD4 count by NRTI agent and HIV RNA suppression including ART initiation year, ART clinic setting, and patient, as random effects in all models (to account for clustering and the longitudinal nature of the data). We additionally assessed for interactions with NRTI that could affect CD4 slope. We completed a time to event analysis for loss-from-care, using a competing risk framework, using death as the competing risk and using robust estimates at site level. We used a random effects Poisson model accounting for site-level effects to calculate incident rates for loss-from-care, adjusted for site-level differences. For mortality, we calculated hazard ratios and adjusted hazard ratios using Cox proportional hazard modeling, controlling for site level effects by using robust estimates with a fixed effect for program (workplace or community). For both single drug substitution and loss-from-care competing risk analyses, we included ART initiation year and ART clinic setting (workplace or community) to adjust for secular and site level effects.

In univariable analysis we assessed for associations between the outcomes and NRTI agent, sex, age, NNRTI agent, WHO clinical stage at ART initiation, CD4 count at ART initiation, HIV RNA at ART initiation, and ART initiation year. Except in the CD4 change analysis, we chose not to use time up-dated data for CD4 count and HIV RNA as these could potentially be affected by NRTI agent characteristics (e.g. tolerability, adherence, potency, etc.) and hence could be on the causal pathway from exposure to outcome.

Because of the considerable imbalance between characteristics between workplace and community programs, we completed sensitivity analyses by repeating all adjusted analyses restricting to either workplace or community sites. We also explored single-drug substitutions in a sensitivity analysis restricting to comparisons between d4T and AZT for the period prior to TDF registration in South Africa, so as to exclude patients who may have been switched from another agent to TDF, not because of adverse events, but because the provider perceived TDF to be superior.

## Results

### Baseline characteristics

**Figure 1:**
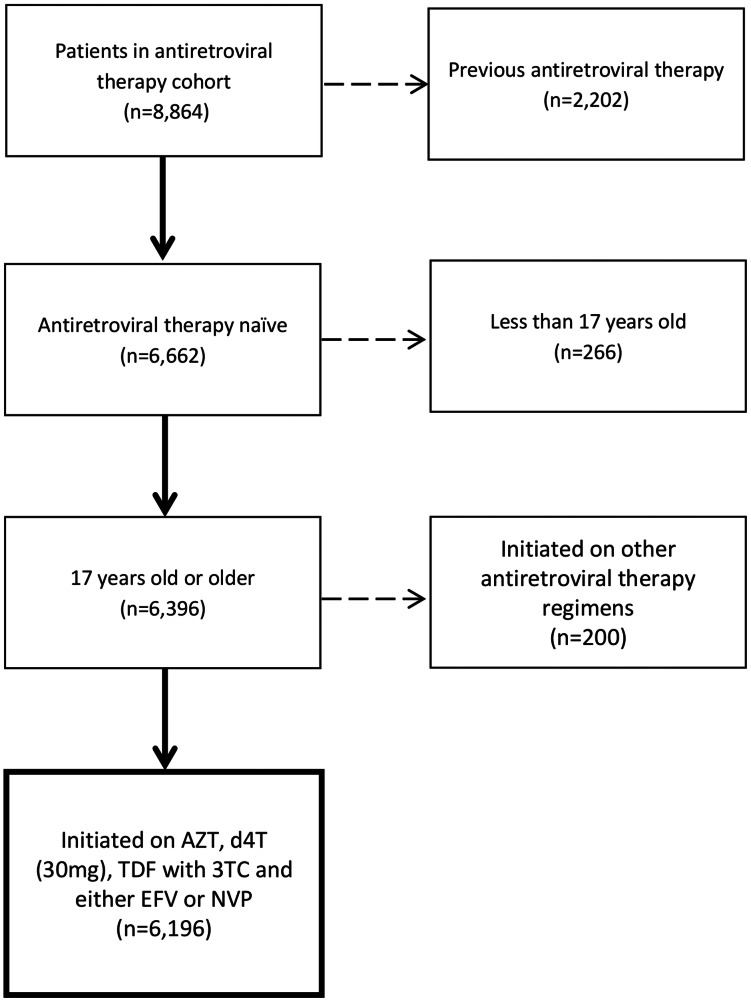
Flow diagram starting with the total number of patients in the ART management cohort, reasons for exclusion, and final sample for this study.

Between June 1, 2007 and June 30, 2009, a total of 8,864 adults initiated ART, of which 6,196 (70.0%) met inclusion criteria. There were 2,668 patients excluded because they were not ART naïve at study entry (n = 2,202), <17 years old (n = 266), or were initiated on a regimen other than the allowed regimens (n = 200; Figure 1). The median age was 40 years (inter-quartile range [IQR]: 34, 47) and median CD4 count prior to ART was 133 cells/mm^3^ (IQR: 66, 193; Table 1). Women comprised 52% of patients. The initial NRTI was TDF for 665 (10.7%), d4T for 4,179 (67.5%), and AZT for 1,352 (21.8%) patients; 3,570 (58%) received EFV and 2,626 (42%) received NVP. NRTI use was not uniformly distributed across clinics; patients initiating TDF and AZT were more likely to be in the workplace program, as were most of the men and more patients with WHO clinical stage I or II disease. Patients initiated on d4T were more likely to be women with lower CD4 counts (Table 1). Total follow-up time was 9,229 person-years and the median follow-up time on ART was 1.9 years (IQR: 1.0–2.0); 1,610 patients died and 500 were lost-from-care.

**Table 1:** Cohort characteristics at ART initiation by nucleoside reverse transcriptase inhibitor.

Characteristic		TDF (n = 665)	D4T (n = 4,179)	AZT (n = 1,352)	p-value
**Sex, n (%)**					<0.001
	Male	527 (79.3)	1,524 (36.5)	920 (68.1)	
	Female	138 (20.7)	2,655 (63.5)	432 (31.9)	
**Age in years, n (%)**					<0.001
	17–29	37 (5.6)	453 (10.8)	96 (7.1)	
	30–39	253 (38.1)	1,733 (41.5)	449 (33.2)	
	40–49	204 (30.7)	1,326 (31.7)	462 (34.2)	
	≥50	171 (25.7)	667 (16.0)	345 (25.5)	
**Initiation Year, n (%)**					<0.001
	2007	22 (3.3)	1,578 (37.8)	586 (43.3)	
	2008	327 (49.2)	1,739 (41.6)	503 (37.2)	
	2009	316 (47.5)	862 (20.6)	263 (19.5)	
**CD4 count at ART initiation, cells/mm^3^, n (%)**					<0.001
	<50	59 (8.9)	660 (15.8)	131 (9.7)	
	50–99	66 (9.9)	616 (14.7)	166 (12.3)	
	100–250	240 (36.1)	1,585 (37.9)	544 (40.2)	
	>250	94 (14.1)	131 (3.1)	157 (11.6)	
	Missing	206 (31.0)	1,187 (28.4)	354 (26.2)	
	Median (IQR)	177 (94, 241)	119 (57, 178)	158 (87, 228)	
**HIV RNA at ART initiation, c/mL, n (%)**					<0.001
	<1000	4 (0.6)	36 (0.9)	18 (1.3)	
	1000–50000	159 (23.9)	1,173 (28.1)	461 (34.1)	
	50001–100000	94 (14.1)	505 (12.1)	170 (12.6)	
	>100000	211 (31.7)	1,068 (25.6)	298 (22.0)	
	Missing	197 (29.7)	1,397 (33.3)	405 (30.0)	
	Log_10_ Median (IQR)	4.9 (4.5, 5.4)	4.8 (4.4, 5.3)	4.7 (4.2, 5.1)	
**WHO clinical stage, n (%)**					<0.001
	I & II	515 (77.4)	1,689 (40.4)	855 (63.2)	
	III & IV	134 (20.2)	2,183 (52.2)	451 (33.4)	
	Missing	16 (2.4)	307 (7.4)	46 (3.4)	
**NNRTI, n (%)**					<0.001
	EFV	590 (88.7)	1,923 (46.0)	1,057 (78.2)	
	NVP	75 (11.3)	2,256 (54.0)	295 (21.8)	
**HIV Program, n (%)**					<0.001
	Workplace	598 (89.9)	102 (2.4)	809 (59.8)	
	Community	67 (10.1)	4,077 (97.6)	543 (40.2)	

AZT: Zidovudine; d4T: Stavudine; TDF: Tenofovir; IQR: Interquartile Range; NNRTI: non-nucleoside reverse transcriptase inhibitor; EFV: Efavirenz; NVP: Nevirapine.

### Single drug substitutions

Over the first two years of ART, 10 patients had a single drug substitution from TDF (1.3 per 100 person-years [PYRs]; 95% confidence interval [CI]: 0.6–2.9), 691 from d4T (8.2 per 100 PYRs; 95% CI: 5.1–13.1), and 95 from AZT (5.1 per 100 PYRs; 95% CI: 3.2–8.5). However, the rate of single drug substitution varied by time on ART. During the first 6 months of ART, the rate of single drug substitution was lowest for TDF (2.6 per 100 PYRs; 95% CI: 1.0–6.8) and was similar for d4T (7.4 per 100 PYRs; 95% CI: 4.5–12.0) and AZT (8.7 PYRs; 95% CI: 5.2–14.7). However, from 6 months onward, the rate was highest for d4T (5.9 per 100 PYRs; 95% CI: 3.3–10.2) and lower for TDF (0.6 per 100 PYRs; 95% CI: 0.2–1.8) and AZT (3.1 per 100 PYRs; 95% CI: 1.7–5.6).

In multivariable modeling, the differences in single agent substitution by time on ART remained (Table 2). During the first 6 months of ART, compared to TDF, the hazard ratio of substitution for d4T was 2.3 (95% CI: 0.27, 19) and for AZT was 5.2 (95% CI: 1.1, 23); a statistically significant difference between AZT and TDF. After 6 months of ART, compared to TDF, the hazard of substitution was 10 (95% CI: 5.8–18) for d4T and 4.4 (95% CI: 2.5, 7.8) for AZT. The change between the first six months and six to 24 months was significant (p for effect modification <0.001). Sex, age, HIV RNA at ART initiation, WHO clinical stage at ART initiation, and ART initiation year were all associated with single agent substitution in the multivariable analysis (Table 2). NNRTI agent was associated with hazard of single-drug substitution in the multivariable analysis, but there was no interaction between NNRTI agent and NRTI. Nor were there interactions between NRTI and either sex or age in predicting single drug substitution.

**Table 2:** Competing-risk regression model for single agent substitution.

		Univariable hazard ratio		Multivariable hazard ratio	
		HR (95% CI)	p- value	aHR* (95% CI)	p- value
**NRTI**					
**0**–**6 months on ART**					
	TDF	Referent	<0.001	Referent	<0.001
	d4T	3.3 (0.41, 25)		2.3 (0.27, 19)	
	AZT	5.3 (1.7, 16)		5.2 (1.1, 23)	
**6**–**24 months on ART**					
	TDF	Referent	<0.001	Referent	<0.001
	d4T	14 (7.7, 27)		10 (5.8, 18)	
	AZT	4.4 (2.8, 7.0)		4.4 (2.5, 7.8)	
**NNRTI**					
	EFV	Referent	0.1	Referent	<0.001
	NVP	1.4 (0.9, 2.3)		0.86 (0.82, 0.90)	
**Sex**					
	Male	Referent	<0.001	Referent	<0.001
	Female	2.5 (1.7, 3.7)		2.2 (2.1, 2.3)	
**CD4 count, at ART initiation, cells/mm^3^**					
	<50	Referent	0.02	Referent	<0.001
	50–100	0.9 (0.72, 1.2)		1.0 (0.8, 1.2)	
	101–250	1.0 (0.76, 1.3)		1.1 (1.0, 1.1)	
	>250	0.44 (0.22, 0.87)		0.65 (0.50, 0.85)	
**HIV RNA at ART initiation, c/mL**					
	<50,000	Referent	0.03	Referent	0.2
	50,000–100,000	0.8 (0.7, 0.9)		0.85 (0.70, 1.1)	
	>100,000	0.9 (0.8, 0.9)		0.92 (0.83, 1.0)	

* also adjusted for year of ART initiation, program and site, age and WHO stage.

CI: Confidence Interval; AZT: Zidovudine; d4T: Stavudine; TDF: Tenofovir; NNRTI: non-nucleoside reverse transcriptase inhibitor; EFV: Efavirenz; NVP: Nevirapine; HR: Hazard Ratio; aHR: adjusted Hazard Ratio.

When restricting to workplace sites for a sensitivity analysis, the adjusted hazard ratio compared to TDF, was 13.8 (95% CI: 3.5–55.1) for d4T and 4.7 (95% CI: 1.3–17.0) for AZT during the first 6 months of ART; after 6 months, the hazard ratio was 9.2 (95% CI: 2.2–38.5) for d4T and 4.7 (95% CI: 1.4–15.8) for AZT. In the community program, when compared to TDF, during the first 6 months of ART, the adjusted hazard ratio for d4T was 0.9 (95% CI: 0.3–3.0) and for AZT was 1.5 (95% CI: 0.4–5.1) while after 6 months the adjusted hazard ratio for d4T was 7.3 (95% CI: 1.8–29.9) and for AZT was 4.2 (95% CI: 1.1–17.8).

We also repeated the analysis restricting to the time period prior to TDF availability, limiting single drug substitutions to AZT from d4T or to d4T from AZT. During the first 6 months, the rate of substitution from d4T was 5.9 per 100 PYRs and from AZT was 5.8 per 100 PYRs. After 6 months the rate from d4T was 4.5 per 100 PYRs and from AZT was 0.5 per 100 PYRs. The hazard ratio for single agent substitution from d4T (compared to AZT) during the first 6 months was 1.2 (95% CI: 0.8–2.0), and from d4T (compared to AZT) from 6–24 months was 7.8 (95% CI: 4.1–14.8).

### Virological suppression at 24 months

The distribution of patients with HIV RNA data at 24 months was 46% of TDF recipients, 36% of d4T recipients, and 42% of AZT recipients. Where HIV RNA data were available, HIV RNA suppression (<400 copies/ml) was achieved in 74% on TDF, 83% on d4T, and 70% of patients on AZT. In the random-effects logistic regression model, when compared to TDF, the odds ratio for virological suppression on d4T was 1.1 (95% CI: 0.7–1.8) and on AZT was 0.8 (95% CI: 0.5–1.1; Table 3). Factors statistically associated with viral suppression in the multivariable model included NNRTI, sex, and CD4 count at ART initiation. There was no interaction between NRTI and NNRTI.

**Table 3:** Random-effects logistic regression model of HIV RNA suppression at 24 months on antiretroviral therapy.

		Univariable odds ratio		Multivariable odds ratio	
		OR (95% CI)	p- value	aOR* (95% CI)	p- value
**NRTI**					
	TDF	Referent		Referent	0.05
	D4T	0.9 (0.6, 1.5)	0.8	1.1 (0.7, 1.8)	
	AZT	0.7 (0.5, 0.9)	0.02	0.8 (0.5, 1.1)	
**NNRTI**					
	EFV	Referent	0.003	Referent	<0.001
	NVP	0.7 (0.6, 0.9)		0.6 (0.4, 0.8)	
**Sex**					
	Male	Referent	0.9	Referent	0.03
	Female	1.0 (0.8, 1.3)		1.3 (1.1, 1.8)	
**CD4 count at ART initiation, cells/mm^3^**					
	<50	Referent	0.08	Referent	0.03
	50–100	1.0 (0.7, 1.5)		1.1 (0.7, 1.6)	
	101–250	1.3 (0.9, 1.9)		1.4 (1.1, 2.0)	
	>250	0.9 (0.6, 1.4)		0.9 (0.6, 1.4)	
**HIV RNA at ART initiation, c/mL**					
	<50,000	Referent	<0.001	Referent	1.0
	50,000–100,000	1.0 (0.7, 1.3)		1.0 (0.7, 1.4)	
	>100,000	1.1 (0.8, 1.4)		1.1 (0.8, 1.4)	

* also adjusted for year of ART initiation, program and site, and age.

aOR: Adjusted Odds Ratio; CI: Confidence Interval; AZT: Zidovudine; d4T: Stavudine; TDF: Tenofovir; NNRTI: Non-Nucleoside Reverse Transcriptase Inhibitor; EFV: Efavirenz; NVP: Nevirapine.

In a sensitivity analysis of HIV RNA suppression, when restricting to workplace sites, the adjusted hazard ratio with TDF as a referent was 1.6 (95% CI: 0.7–3.9) for d4T and 0.9 (95% CI: 0.6–1.6) for AZT. Restricting to the community program, the adjusted hazard ratio with TDF as the referent agent was 1.5 (95% CI: 0.4–5.6) for d4T and was 1.2 (95% CI: 0.3–4.8) for AZT.

### CD4 change to 24 months

The distribution of patients with over 12 months of CD4 data for inclusion in assessing CD4 change was 74% of TDF recipients, 62% of d4T recipients, and 73% of AZT recipients. In mixed linear regression, with NRTI agent as the only fixed effect, NRTI agent was significantly associated with CD4 increase with an annual increase for TDF, d4T, and AZT of 67.0 (95% CI: 61.2, 72.8), 79.2 (95% CI: 76.9, 81.6), and 53.1 (95% CI: 49.2, 56.9) cells/mm^3^/year, respectively (p<0.001; Table 4). In adjusted multivariable analysis, in which we included time updated HIV RNA suppression and sex as modifiers to the slope along with sex, age, HIV RNA at ART initiation, and NNRTI as terms in the intercept, a significant association was maintained. For time points with HIV RNA suppression, the increase in CD4 count was 83.9 (95% CI: 78.0, 90.0) for TDF, 83.0 (95% CI: 80.0, 86.0) for d4T, and 73.0 cells/mm^3^/year (95% CI: 68.0, 76.6) for AZT (p<0.001).

**Table 4:** CD4 count slope based on mixed linear regression model during the first 24 months of ART.

		Unadjusted, cells/mm^3^/year (95% confidence interval)	P for difference	Adjusted*, cells/mm^3^/year (95% confidence interval)	P for difference
**NRTI agent**			<0.001		<0.001
	TDF	67.0 (61.2, 72.8)		83.9 (78.0, 90.0)	
	D4T	79.2 (76.9, 81.6)		83.0 (80.0, 86.0)	
	AZT	53.1 (49.2, 56.9)		73.0 (68.0, 76.6)	

NRTI: nucleoside reverse transcriptase inhibitor; TDF: tenofovir difumarate; AZT: zidovudine.

* slope adjusted for sex and HIV viral suppression; intercept adjusted for sex, age, NNRTI, and baseline HIV RNA.

In a sensitivity analysis, when restricting to workplace sites, we continued to observe a statistically significant association between NRTI agent and CD4 slope (p<0.001) with a slope for TDF of 85.3 (95% CI: 78.4, 92.3), for d4T of 90.0 (95% CI: 77.6, 102.3), and for AZT of 69.0 (95% CI: 62.5, 75.4) cells/mm^3^/year. In the community program, the association was no longer statistically significant (p = 0.3): TDF, 65.2 (95% CI: 42.6, 87.7); d4T, 82.7 (95% CI: 79.3, 86.1); and AZT, 78.4 (71.6, 85.6).

### Loss-from-care

During the 24 month follow-up period, the rate for loss-from-care appeared similar for TDF (9.8 per 100 PYRs; 95% CI: 4.2–23.0) and AZT (9.5 per 100 PYRs; 95% CI: 4.1–21.7) and was highest for patients on d4T (11.8 per 100 PYRs; 95% CI: 5.1–27.5). In the adjusted model, compared to TDF, the hazard ratio for d4T was 1.5 (95% CI: 1.1–1.9) and for AZT was 1.2 (95% CI: 1.1–1.4; Table 5), a significantly higher loss for d4T and AZT than TDF.

**Table 5:** Competing-risk regression model for loss-from-care.

		Univariable hazard ratio		Multivariable hazard ratio	
		HR (95% CI)	p- value	aHR* (95% CI)	p- value
**NRTI**					
	TDF	Referent	<0.001	Referent	0.008
	d4T	1.7 (1.3, 2.2)		1.5 (1.1, 1.9)	
	AZT	1.1 (0.9, 1.5)		1.2 (1.1, 1.4)	
**NNRTI**					
	EFV	Referent	0.01	Referent	0.06
	NVP	0.5 (0.3, 0.9)		0.8 (0.7, 1.0)	
**Sex**					
	Male	Referent	0.004	Referent	<0.001
	Female	0.4 (0.2, 0.8)		0.8 (0.7, 0.9)	
**CD4 at ART initiation, cells/mm^3^**					
	<50	Referent	<0.001	Referent	<0.001
	50–100	1.0 (0.7, 1.4)		1.0 (0.7, 1.4)	
	101–250	1.3 (0.9, 2.1)		1.1 (0.9, 1.5)	
	>250	1.7 (1.3, 2.3)		1.2 (1.1, 1.4)	
**HIV RNA at ART initiation, c/mL**					
	<50,000	Referent	<0.001	Referent	<0.001
	50,001–100,000	1.0 (0.9, 1.1)		1.0 (0.9, 1.2)	
	>100,000	0.9 (0.8, 1.1)		1.0 (0.8, 1.2)	

* also adjusted for program and site, initiation year and age.

CI: Confidence Interval; AZT: Zidovudine; d4T: Stavudine; TDF: Tenofovir; EFV: Efavirenz; NVP: Nevirapine; HR: Hazard Ratio; aHR: Adjusted Hazard Ratio.

In the sensitivity analysis when restricting to workplace sites, the hazard ratio for loss-from-care for d4T was 0.8 (95% CI: 0.4, 1.7) and for AZT was 1.1 (95% CI: 0.8, 1.5) when compared to TDF. When restricting to community sites, the hazard ratio for d4T was 2.0 (95% CI: 0.8, 5.0) and for AZT was 1.4 (95% CI: 0.5, 3.6) when compared to TDF. Thus, when analyzed separately by workplace and community clinics, NRTI agent was not significantly associated with loss-from-care. There were no significant interactions between loss-from-care and duration on ART, nor were there interactions between NRTI and any of sex, age, or NNRTI in predicting loss-from-care.

### Mortality

The overall adjusted mortality rate for TDF was 9.2 per 100 PYRs (95% CI: 5.9, 14.4), for d4T was 17.8 per 100 PYRs (95% CI: 11.9, 26.4), and for AZT was 11.1 per 100 PYRs (95% CI: 7.3, 16.7). In adjusted proportional hazards modeling, the hazard ratio was 2.7 (95% CI: 2.0, 3.6) for d4T and 1.4 (95% CI: 1.3, 1.5) for AZT when compared to TDF (Table 6).

**Table 6:** Cox-proportional hazard model for mortality.

		Univariable hazard ratio		Multivariable hazard ratio	
		HR (95% CI)	p- value	aHR* (95% CI)	p- value
**NRTI**					
	TDF	Referent	<0.001	Referent	<0.001
	d4T	2.7 (2.0, 3.7)		2.7 (2.0, 3.6)	
	AZT	1.4 (1.3, 1.5)		1.4 (1.3, 1.5)	
**NNRTI**					
	EFV	Referent	0.6	Referent	0.5
	NVP	1.1 (0.8, 1.6)		1.0 (0.9, 1.0)	
**Sex**					
	Male	Referent	0.4	Referent	0.8
	Female	1.2 (0.7, 1.9)		1.0 (0.9, 1.1)	
**CD4 count at ART initiation, cells/mm^3^**					
	<50	Referent	<0.001	Referent	<0.001
	50–100	0.9 (0.7, 1.0)		0.9 (0.8, 1.0)	
	101–250	0.6 (0.5, 0.7)		0.7 (0.6, 0.7)	
	>250	0.4 (0.2, 0.9)		0.6 (0.4, 0.9)	
**HIV RNA at ART initiation, c/mL**					
	<50,000	Referent	<0.001	Referent	<0.001
	50,000–100,000	1.3 (1.1, 1.4)		1.2 (1.0, 1.3)	
	>100,000	1.4 (1.3, 1.5)		1.2 (1.2, 1.3)	

* also adjusted for year of ART initiation, program, site, age and WHO stage.

HR: Hazard Ratio; aHR: Adjusted Hazard Ratio; CI: Confidence Interval; AZT: Zidovudine; d4T: Stavudine; TDF: Tenofovir; NNRTI: non-nucleoside reverse transcriptase inhibitor; EFV: Efavirenz; NVP: Nevirapine.

In a sensitivity analysis, when restricting to workplace sites, the adjusted hazard ratio for d4T was 1.5 (95% CI: 1.1–2.2) and for AZT was 1.2 (95% CI: 0.9–1.4) with TDF as a referent. In the community program the adjusted hazard ratio for d4T was 1.5 (95% CI: 1.3–1.8) and for AZT was 1.0 (95% CI: 0.7–1.5) with TDF as the referent agent. Thus d4T was associated with higher mortality in both program types.

## Discussion

During the first 24 months of ART, use of TDF as part of a first-line regimen was associated with lower rates of single drug substitution than either 30 mg of d4T or AZT. In addition, mortality appeared lower for TDF and AZT than for d4T. However, among patients alive and in-care, HIV RNA suppression was similar and loss-from-care was not consistently different by NRTI agent while CD4 count recovery was attenuated for AZT.

An important strength of our analysis is that our study population received care in a large routine HIV care program. Furthermore, we had robust death ascertainment through linkage to a national vital status registry. However, use of data from routine service delivery programs also has limitations, including missing data, the potential for secular trends, and possible indication bias. For example, we had incomplete data on suspected adverse reactions. Thus we did not attempt an analysis based on these data and instead used the proxy of single-drug substitution to indicate a possible adverse event. Although single-drug substitutions may have occurred for other reasons, we believe the majority of these substitutions occurred as a result of suspected side effects, as the only reason for a single drug NRTI substitution provided in the treatment guidelines was for side effects. Missing HIV RNA data may have affected our virologic suppression analysis; however, we do not suspect a bias in HIV RNA enumeration by NRTI agent. Another limitation is lack of adherence data; however, such data are difficult to accurately and consistently collect in routine care settings.

Most importantly, confounding by indication is an inherent problem when analyzing cohort data gathered from multiple sites with changes in care over time. We have attempted to address the potential of time-effect bias in our results through adjusting for ART initiation year by either stratification in the Cox proportional hazards analysis or including it as a random effect in the mixed effects models. More importantly, there is a substantial risk of bias due to inclusion of patients from workplace and community settings as ART regimens and patients differed considerably between the settings. To address this limitation, we adjusted for program or clinic in all analyses. In addition, we further assessed for marked variation in results through sensitivity analyses limited to either the workplace or community setting. We are reassured by our findings of overall similar direction of effect when restricting the analyses; however, the effect size varied. We believe that our sensitivity analyses help to validate the overall direction of associations, although the specific effect sizes may be imprecise and population dependent. The notable discrepancy was with loss-from-care in which NRTI agent was statistically associated with loss-from-care in the full analysis but lost association in the sensitivity analyses. The reason for this remained unclear even after review of clinic chart abstractions to identify reasons for loss-from-care that may not have been indicated by routinely abstracted data. Further studies of loss-from-care, by ART regimen, from other programs would be of value.

Our study findings were similar, in terms of single drug substitutions, to a study from Zambia comparing TDF, d4T, and AZT [5]. In that study, single drug substitutions occurred most frequently for AZT early after ART initiation; however, over time, single drug substitutions occurred at a higher rate for d4T. However, the study from Zambia reported an adjusted hazard ratio of 1.34, compared to our hazard of 10.6, for substitutions from d4T. The longer follow-up time in our study may have partly contributed to the difference, as d4T toxicity increases with time on ART [10]. A study from Lesotho that had longer follow-up, reported a hazard ratio of substitution from d4T of 5.4 and from AZT of 2.3, when compared to TDF [6]. Another possible reason for a larger effect size in our study is that alternative agents were more readily available in our South African cohort. This may have led to a lower threshold for single drug substitutions from AZT or d4T to TDF than from AZT to d4T or d4T to AZT prior to TDF availability. The higher rates of substitution that occurred in our study after TDF became available support this hypothesis. As a result, it is likely that we have overestimated the frequency of serious adverse events that truly required drug substitution. It is notable that in our study, as well as the studies from Zambia and Lesotho, the d4T regimen used was 30 mg twice daily for all body weights, yet the rates of substitutions from d4T in response to adverse events were still high. The high rate of side effects from 30 mg of d4T has also been reported from a comparison of 30 and 40 mg dosing of d4T [11].

Neither the Zambian nor the Lesotho studies identified differences in loss-from-care by regimen. Drug tolerability is a reported reason for discontinuation of care [12], how important a factor it is in any of these environments is unclear.

Our mortality findings were also similar to the Zambian and Lesotho studies in which d4T was associated with an increased mortality hazard. Both AZT and d4T were associated with a higher mortality in the Lesotho study [6]. Of note, our total overall mortality was high. This finding may be related to increased ascertainment through the use of linkage to a vital statistics register [9], [13]–[16].

We did not observe a difference in HIV RNA suppression by NRTI at 24 months. The comparable studies from Zambia and Lesotho did not assess this outcome as HIV RNA enumeration was not part of routine ART care in those countries. This finding suggests that among patients remaining in-care and attending clinic sessions, agent efficacy was similar. In addition, there was no evidence that the worse outcomes with d4T were mediated through a lower rate of HIV RNA suppression (or adherence as estimated by HIV RNA suppression).

Although HIV RNA suppression did not differ by NRTI, CD4 count slope was slightly less for patients receiving AZT. This is consistent with prior AZT experience, although the absolute difference in slope is less pronounced than reported from several clinical trials in which the differences were approximately 30 cells/mm^3^ [17]–[20]. The clinical implications of a slightly slower CD4 count rise with AZT are unclear.

In our cohort, TDF appeared to outperform d4T at the 30 mg dosing and AZT in terms of need for drug substitution and all-cause mortality. From a public health standpoint, fewer drug substitutions may be important for program success and controlling costs. We believe that our results add to the data supporting the public health use of TDF as part of a first-line regimen, as recommended by the WHO. Our findings also suggest that, even at the current lower dose of d4T, the agent continues to have adverse effects leading to single-drug substitutions and may be contributing to increased losses from care and mortality. Longer-term evaluations of these regimens are needed.
